# Cytomegalovirus reactivation in immunocompetent mechanical ventilation patients: a prospective observational study

**DOI:** 10.1186/s12879-021-06698-0

**Published:** 2021-09-30

**Authors:** Zhihui Zhang, Xuesong Liu, Ling Sang, Sibei Chen, Zhan Wu, Jierong Zhang, Yining Sun, Yongbo Huang, Yonghao Xu, Weiqun He, Yimin Li, Xiaoqing Liu

**Affiliations:** 1 State Key Laboratory of Respiratory Diseases, National Clinical Research Center for Respiratory Disease, Guangzhou Institute of Respiratory Health, Department of Critical Care Medicine, The First Affiliated Hospital of Guangzhou Medical University, Guangzhou, Guangdong 510120 People’s Republic of China; 2 Guangzhou Medical University, Guangzhou, Guangdong 511436 People’s Republic of China

**Keywords:** Cytomegalovirus reactivation, Immunocompetent, Critically ill, Epidemiology, Predictors

## Abstract

**Background:**

Cytomegalovirus (CMV) reactivation is associated with adverse prognoses of critically ill patients. However, the epidemiology and predictors of CMV reactivation in immunocompetent patients receiving mechanical ventilation (MV) are not clear. The aim of this study was to investigate the epidemiology and predictors of CMV reactivation in immunocompetent patients requiring MV.

**Methods:**

A single-center, prospective observational study (conducted from June 30, 2017 to July 01, 2018) with a follow-up of 90 days (September 29, 2018) that included 71 CMV-seropositive immunocompetent patients with MV at a 37-bed university hospital general intensive care unit (ICU) in China. Routine detection of CMV DNAemia was performed once a week for 28 days (Days 1, 7, 14, 21, and 28). CMV serology, laboratory findings, and clinical data were obtained during hospitalization.

**Results:**

Among 71 patients, 13 (18.3%) showed CMV reactivation within 28 days in the ICU. The median time to reactivation was 7 days. CMV reactivation was related to various factors, including body mass index (BMI), sepsis, N-terminal pro-b-type natriuretic peptide (NT-proBNP), blood urea nitrogen (BUN), and hemoglobin (Hb) levels (P < 0.05). In the multivariate regression model, BMI, Hb level, and sepsis were independently associated with CMV reactivation patients (P < 0.05). Moreover, the area under the receiver operating characteristic (AUROC) of BMI, Hb, and BMI combined with Hb was 0.69, 0.70, and 0.76, respectively. The duration of MV, hospitalization expense, length of ICU stay, and 90 day all-cause mortality rate in patients with CMV reactivation was significantly higher than in those without CMV reactivation (P < 0.05).

**Conclusions:**

Among immunocompetent patients with MV, the incidence of CMV reactivation was 18.3%. CMV reactivation was associated with several adverse prognoses. BMI, Hb, and sepsis were independent risk factors for CMV reactivation. BMI and Hb may predict CMV reactivation.

**Supplementary Information:**

The online version contains supplementary material available at 10.1186/s12879-021-06698-0.

## Introduction

In the general population, the positive seroprevalence for cytomegalovirus (CMV) is as high as 83% [1]. Primary infection usually occurs during pregnancy or childhood, and the infection rate is related to several factors, such as location and health care availability [2]. When the body is infected with CMV, it will carry it throughout life [2, 3]. CMV infection status is a latent infection, but reactivation occurs under certain conditions, leading to active infection.

The immune function is closely related to the occurrence of CMV reactivation. CMV can reactivate in the course of diminished immunity and frailty [3, 4]. Critically ill patients have severe diseases (such as sepsis, burns, and acute respiratory distress syndrome) and impaired immune function [5–7]. CMV is prone to reactivation in ICU patients [2, 4]. In recent decades, numerous studies have suggested that the incidence of CMV reactivation in critically ill immunocompetent patients is 9–71% [8]. Moreover, CMV reactivation is associated with various adverse clinical outcomes, such as prolonged mechanical ventilation (MV), extracorporeal membrane oxygenation (ECMO) duration, increased length of hospitalization, and mortality [7–9]. However, CMV reactivation is challenging to predict earlier because it lacks particular clinical manifestations. At present, CMV reactivation is mainly diagnosed by measuring the viral load [2, 10]. Some studies have shown that CMV reactivation may be associated with sepsis, transfusion, and cytokine levels. Still, none of them screened out effective indicators to predict CMV reactivation [4, 5, 8, 11]. Therefore, there is an urgent need to find indicators that can effectively predict CMV reactivation and further study the epidemiology of CMV reactivation in immunocompetent patients requiring MV.

## Methods

### Setting

This study was conducted in the general ICU of the First Affiliated Hospital of Guangzhou Medical University, a national teaching hospital with 2000 beds; the ICU has 37 independent beds. The study was given official approval by the Ethics Committee of the First Affiliated Hospital of Guangzhou Medical University and authorized by the Chinese Clinical Trial Registry [No. ChiCTR-ROC-17013296 (2017/11/8)]. Written informed consent was obtained from patients or authorized surrogates.

### Patients

From June 30, 2017 to July 1, 2018, consecutive mechanically ventilated patients cared for in the ICU were screened. Patients were eligible unless they met the following exclusion criteria: (1) Inability to provide informed consent; (2) Age < 18 years; (3) Pregnant or lactation; (4) Survival time < 72 h; (5) Readmitted to ICU; (6) CMV seronegative; (7) Required invasive mechanical ventilation (IMV) before admission or did not need IMV after admission; (8) Received antiviral drugs before admission; (9) Diagnosed with solid organ or bone marrow tumor; (10) Neutropenic (white cell counts < 1000/uL or neutrophils < 500/uL); (11) Systemic glucocorticoids were used (prednisone > 0.1 mg/kg for > 3 months, methylprednisolone > 40 mg/d for > 1 week, or equivalent); (12) Diagnosed immunodeficiency (transplantation, HIV, or immunosuppressive drugs); (13) Post-surgical patients transferred to ICU for monitoring.

### Study design

Screening of all patients admitted to ICU from June 30, 2017 to July 1, 2018. Patients who met the exclusion criteria were excluded from the study. Data are regularly recorded until the subject is discharged from ICU or general ward (or death). On September 29, 2018, the study ended (a follow-up of 90 days) and patients were followed through telephone follow-up. Routine detection of CMV DNAemia once a week for 28 days (Days 1, 7, 14, 21, and 28), CMV serology, laboratory findings, and clinical data were obtained during hospitalization. Furthermore, patients were divided into a reactivation group (CMV DNAemia ≥ 500 copies/mL) and a non-reactivation group (CMV DNAemia < 500 copies/mL).

### Procedures and data collection

Two trained researchers performed CMV DNAemia testing, and the results were recorded on electronic records. The clinical data from 71 cases, including patient demographics, clinical symptoms and signs, laboratory findings, and clinical outcomes, were extracted from the electronic records by two independent intensivists who subsequently cross-checked the data for accuracy. A third independent reviewer resolved the disagreement. All data were entered into the computerized database for further statistical analyses.

### Study definitions

CMV serology (anti-CMV IgG) was determined in a plasma sample obtained 24 h within ICU admission (Human Anti-Cytomegalovirus IgG, Abcam Products, Cambridge, United Kingdom). Subsequently, once a week for 28 days (Days 1, 7, 14, 21, and 28), real-time Taqman CMV DNA polymerase chain reaction was used to determine viral load in seropositive patients. Viral load values were calibrated to the CMV World Health Organization Standard (CMV reactivation was defined as a load greater than or equal to 500 copies/mL). Screening for CMV serology or viral load was part of routine clinical practice in this hospital. Both CMV serology and reactivation results from this study were made available to the treating physicians. However, it was up to the attending physician to decide whether the patient should be treated.

### Statistical analysis

Continuous variables were expressed as mean ± SD or median (interquartile ranges, IQRs) and compared with the Wilcoxon rank-sum test. Categorical variables were expressed as counts and percentages, and compared using the chi-square test or Fisher’s exact test as appropriate. The risk factors for CMV reactivation were screened using a univariate logistic regression model. Variables with a P-value of 0.05 or less were considered the potential risk factors and further imported into the multivariate logistic regression analysis. The CMV reactivation risk model was established by calculating the regression coefficient (β), odds ratio (OR), and 95% confidence interval (CI). The receiver operating characteristic (ROC) curve was used to evaluate the predictive value of CMV reactivation. The area under the ROC curve (AUC), 95% CI, P-value, cut-off, sensitivity, and specificity were calculated. Kaplan–Meier (KM) survival analysis was used to compare the 90 day survival rate between the two groups, and the log-rank test was used to compare the survival curve and hazard ratio (HR). The significance threshold was set at a two-sided P-value of less than 0.05. All statistical analyses or charting were performed using SPSS version 25.0 (SPSS Inc., USA) and GraphPad Prism 8.0 (Graphpad Software Inc., USA).

## Results

### Incidence of CMV reactivation

**Fig. 1:**
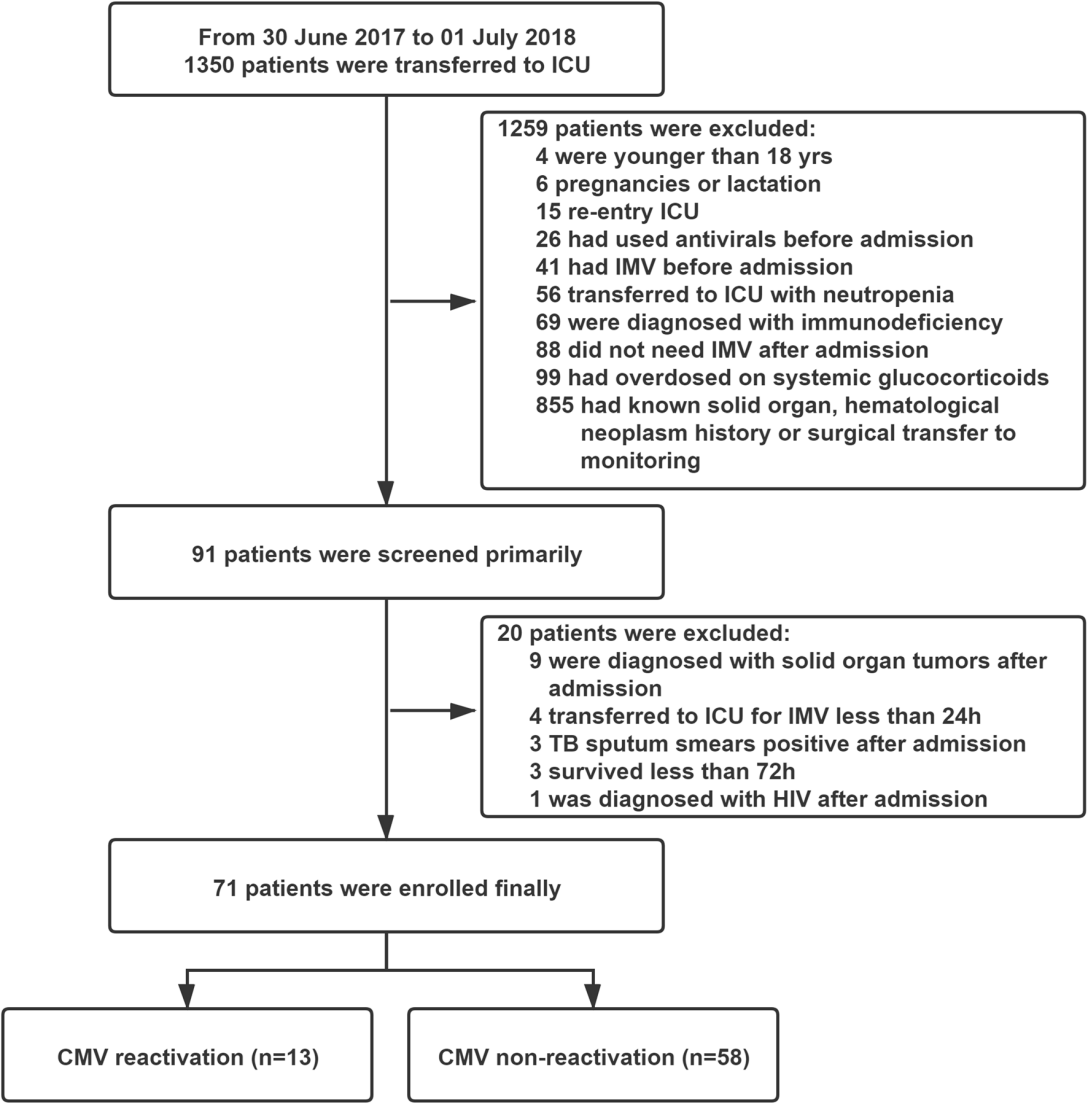
Flowchart of Patient Enrollment

A total of 1,350 patients were admitted to ICU during the study period. As shown in Fig. 1, 91 patients were initially screened for inclusion in the study, and 20 were excluded because of solid organ tumors (n = 9), IMV less than 24 h (n = 4), sputum smears positive after admission (n = 3), death or discharged within 72 h of admission (n = 3), and HIV (n = 1). Eventually, 71 patients were enrolled.

Surprisingly, the study revealed that CMV seropositivity was observed to be 100% during patients screening. Among the 71 enrolled patients, there were 13 cases of CMV reactivation within a 28 day ICU entry, corresponding to an incidence of CMV reactivation of 18.3% (shown in Additional file 1: Figure S1). Of 13 patients with CMV reactivation, 6 (46.1%) were reactivated within 24 h after entering ICU, 1 (7.7%) on Day-7, 4 (30.8%) on Day-14, 1 (7.7%) on Day-21, and 1 (7.7%) on Day-28 (shown in Additional file 1: Figure S2).

### Clinical characteristics

The clinical characteristics of the study population at the time of ICU admission are shown in Table 1. Among 71 patients with complete data, more than 80% were male. The mean age was 64 ± 14 years. The single most striking observation to emerge from the data comparison was that patients with CMV reactivation had lower body weight [mean: 51.1 ± 10.6 vs. 61.6 ± 11.6 (kg), P = 0.01] and body mass index (BMI) [mean: 19.9 ± 4.1 vs. 22.1 ± 3.4 (kg/m^2^), P = 0.03] than the non-reactivation group. There were also differences in sepsis between CMV reactivation and non-CMV reactivation [53.8% vs. 19.0% (n), P = 0.02]. There were, however, no significant differences in other clinical characteristics between CMV reactivation and non-reactivation groups. From the data in Table 2, patients with CMV reactivation had markedly higher levels of N-terminal pro-b-type natriuretic peptide (NT-proBNP) [median: 7745 vs. 1805 (pg/mL), P = 0.01], blood urea nitrogen (BUN) [median: 13.6 vs. 8.6 (mmol/L), P = 0.03)] and hemoglobin (Hb) [median: 98.0 vs. 104.0 (g/L), P = 0.03]. Nevertheless, there were no statistically evident differences for immune indicators between the two groups (shown in Additional file 1: Table S1).

**Table 1:** Clinical characteristics of the study patients

	Overall	CMV reactivation		
	N = 71	Yes (n = 13, 18.3%)	No (n = 58, 81.7%)	*P*
Age (yr)	64 ± 14	68 ± 12	63 ± 14	0.20
Gender, n (%)				0.37
Male	58 (81.7)	9 (69.2)	49 (84.5)	-
Female	13 (18.3)	4 (30.8)	9 (15.5)	-
Height (cm)	168 (160–171)	160 (160–167)	168 (160–172)	0.10
Weight (kg)^*a*^	**58.5 ± 11.8**	**51.1 ± 10.6**	**61.6 ± 11.6**	**0.01**
BMI (kg/m^2^) ^*a*^	**21.3 ± 3.6**	**19.9 ± 4.1**	**22.1 ± 3.4**	**0.03**
Severity of Score				
APACHE II	20 ± 7	22 ± 8	20 ± 7	0.37
SOFA	8 (5–11)	8 (5–10)	8 (6–11)	0.80
Comorbidities, n (%)				
Hypertension	34 (47.9)	6 (46.2)	28 (48.3)	0.89
COPD	28 (39.4)	6 (46.2)	22 (37.9)	0.58
Coronary Heart Disease	19 (26.8)	4 (30.8)	15 (25.9)	0.99
Diabetes	18 (25.4)	2 (15.4)	16 (27.6)	0.58
Chronic Kidney Disease	6 (8.5%)	1 (7.7)	5 (8.6)	NA
Bronchiectasis	8 (11.3)	1 (7.7)	7 (12.1)	NA
Asthma	2 (2.8)	1 (7.7)	1 (1.7)	0.34
Rheumatic Heart Disease	1 (1.4)	0 (0)	1 (1.7)	NA
Complications, n (%)				
Surgical Factors	14 (19.7)	1 (7.7)	13 (22.4)	0.41
Heart Surgery	13 (18.3)	1 (7.7)	12 (20.7)	0.49
Trauma	1 (1.4)	0 (0)	1 (1.7)	NA
Internal Medicine Factors	57 (80.3)	12 (92.3)	45 (77.6)	0.41
Severe Pneumonia	48 (67.6)	10 (76.9)	38 (65.5)	0.64
AECOPD	25 (35.2)	5 (38.5)	20 (34.5)	NA
Sepsis^*a*^	**18 (25.4)**	**7 (53.8)**	**11 (19.0)**	**0.02**
AKI	17 (23.9)	4 (30.8)	13 (22.4)	0.78
Bronchiectasis Infection	8 (11.3)	1 (7.7)	7 (12.1)	NA
ARDS	7 (9.9)	3 (23.1)	4 (6.9)	0.21
Asthma Exacerbation	3 (4.2)	1 (7.7)	2 (3.4)	0.46
Acute Coronary Syndrome	3 (4.2)	0 (0)	3 (5.2)	NA
Heart Valve Disease	2 (2.8)	0 (0)	2 (3.4)	NA
Liver Failure	1 (1.4)	0 (0)	1 (1.7)	NA
Acute Suppurative Cholangitis	1 (1.4)	0 (0)	1 (1.7)	NA

^a^*P* < 0.05; Continuous variables were expressed as Mean ± SD or Median (IQRs); Bold font indicates the difference was statistically significant. *BMI* Body Mass Index, *APACHE II *Acute Physiology and Chronic Health Evaluation, *SOFA* Sequential Organ Failure Assessment, *COPD* Chronic Obstructive Pulmonary Disease, *AECOPD* Acute Exacerbation of Chronic Obstructive Pulmonary Disease, *AKI* Acute Kidney Injury, *ARDS* Acute Respiratory Distress Syndrome

**Table 2:** Vital signs and laboratory findings of the study patients at the time of ICU admission

	Overall	CMV reactivation		
	N = 71	Yes (n = 13, 18.3%)	No (n = 58, 81.7%)	*P*
Basic Vital Signs				
Average Blood Pressure (mmHg)	92 (82–103)	95 (90–103)	92 (80–102)	0.36
Heart Rate (bp)	110 ± 26	115 ± 26	109 ± 27	0.47
Respiratory Rate (t/m)	23 (20–26)	26 (19–26)	23 (20–26)	0.89
Temperature (℃)	36.8 (36.6–37.2)	36.6 (36.6–37.2)	36.9 (36.6–37.2)	0.43
SPO_2_ (%)	97 (95–99)	97 (96–98)	97 (95–99)	0.88
Laboratory Findings				
P/F	234 ± 94	217 ± 76	238 ± 97	0.49
Nt-proBNP (pg/mL)^*a*^	**2499 (611–8207)**	**7745 (2869–19,376)**	**1805 (500–7561)**	**0.01**
AST (U/L)	42.6 (30.0–85.9)	41.0 (28.0–85.9)	42.8 (30.0–81.1)	0.63
ALT (U/L)	25.5 (15.2–53.7)	26.5 (10.2–55.9)	24.4 (15.3–50.5)	0.94
T-BIL(μmol/L)	14.0 (9.7–35.1)	10.9 (9.1–17.2)	15.2 (10.2–39.6)	0.13
Scr (μmol/L)	130 (70–223)	133 (70–205)	128 (70–223)	0.95
BUN (mmol/L)^*a*^	**9.3 (5.7–15.4)**	**13.6 (10.8–20.7)**	**8.6 (5.7–14.1)**	**0.03**
PT (s)	16 (15–17)	16 (15–17)	16 (15–17)	0.82
APTT (s)	41 (36–48)	43 (36–49)	41 (36–47)	0.67
PCT (ng/mL)	0.9 (0.2–7.7)	2.5 (0.3–10.8)	0.9 (0.2–7.4)	0.36
Hypersensitive CRP (mg/L)	107 (32–142)	107 (68–130)	98 (26–142)	0.34
ESR (mm/h)	45 (18–85)	42 (17–75)	45 (18–85)	0.81
G Test (pg/mL)	14.9 (0–47.8)	21.1 (0–56.5)	10.4 (0–45.2)	0.50
GM Test (Aspergillus) (μg/L)	0.33 (0–0.44)	0.32 (0.28–0.37)	0.36 (0–0.44)	0.71
GM Test (Cryptococcus), n (%)	5 (7.0)	0 (0)	5 (8.6)	0.58
White Blood Cells (10^9^/L)	12.5 (8.9–17.1)	14.8 (11.2–20.5)	12.4 (8.9–15.9)	0.28
Neutrophils (10^9^/L)	11.3 (7.6–15.4)	13.3 (10.6–19.4)	11.1 (7.6–14.8)	0.22
Lymphocytes (10^9^/L)	0.4 (0.3–0.9)	0.4 (0.2–0.5)	0.5 (0.3–0.9)	0.19
Monocytes (10^9^/L)	0.7 (0.4–1.0)	0.4 (0.2–0.9)	0.7 (0.4–1.0)	0.16
Eosinophils (10^9^/L)	0 (0–0.01)	0 (0–0.01)	0 (0–0)	0.32
Basophils (10^9^/L)	0 (0–0.02)	0 (0–0.04)	0 (0–0.01)	0.33
Erythrocyte (10^12^/L)	3.40 ± 0.85	3.05 ± 0.79	3.52 ± 0.84	0.07
Hemoglobin (g/L)^*a*^	**102 (82–118)**	**98 (70–102)**	**104 (88–121)**	**0.03**
Platelet (10^9^/L)	177 ± 91	190 ± 116	174 ± 86	0.65

^a^*P* < 0.05. Continuous variables were expressed as Mean ± SD or Median (IQRs); Bold font indicates the difference was statistically significant. *SPO*_*2*_ Arterial oxygen saturation, *P/F (PaO*_*2*_*/FiO*_*2*_*)* the ratio between the arterial partial pressure of oxygen and the inspiratory concentration of oxygen, *Nt-proBNP* N-terminal Pro-B-type Natriuretic Peptide; *AST* Aspartate Aminotransferase, *ALT* Alanine Transaminase, *T-BIL* Total Bilirubin, *Scr* Serum Creatinine, *BUN* Serum Urea Nitrogen; PT: Prothrombin Time; APTT: Activated Partial Thromboplastin Time, *PCT* Procalcitonin, *CRP* C-reactive Protein, *ESR* Erythrocyte Sedimentation Rate

### Risk factors and predictors

Multivariable logistic regression analysis of factors associated with CMV reactivation is shown in Table 3. In the multivariate regression model, lower BMI [OR: 1.25, 95% CI: 1.03–1.53, P = 0.03], lower Hb concentration [OR: 1.04, 95% CI: 1.01–1.08, P = 0.03], and presence of sepsis [OR: 0.10, 95% CI: 0.02–0.50, P < 0.01] were associated with patients with CMV reactivation. Based on the regression coefficient (β), BMI [β: − 0.23] and Hb [β: − 0.04] were protective factors, while sepsis [β: 2.32] was a risk factor. Meanwhile, these results indicated that the risk of CMV reactivation increased by 125% for each 1 kg/m^2^ decrease in BMI level, increased by 104% for each 1 g/L decrease of Hb level, and increased by 10% once sepsis occurred.

**Table 3:** Risk factors for CMV reactivation

Variables	β	Wald	OR	95% CI	*P*
BMI (kg/m^2^)^*a*^	− 0.23	4.95	1.25	1.03–1.53	0.03
Hb (g/L)^*a*^	− 0.04	4.99	1.04	1.01–1.08	0.03
Sepsis^*b*^	2.32	7.74	0.10	0.02–0.50	< 0.01

^a^*P* < 0.05; ^b^*P* < 0.01; *β* Regression Coefficient, *OR* Odds Ratio; *95% CI* 95% Confidence Interval

**Fig. 2:**
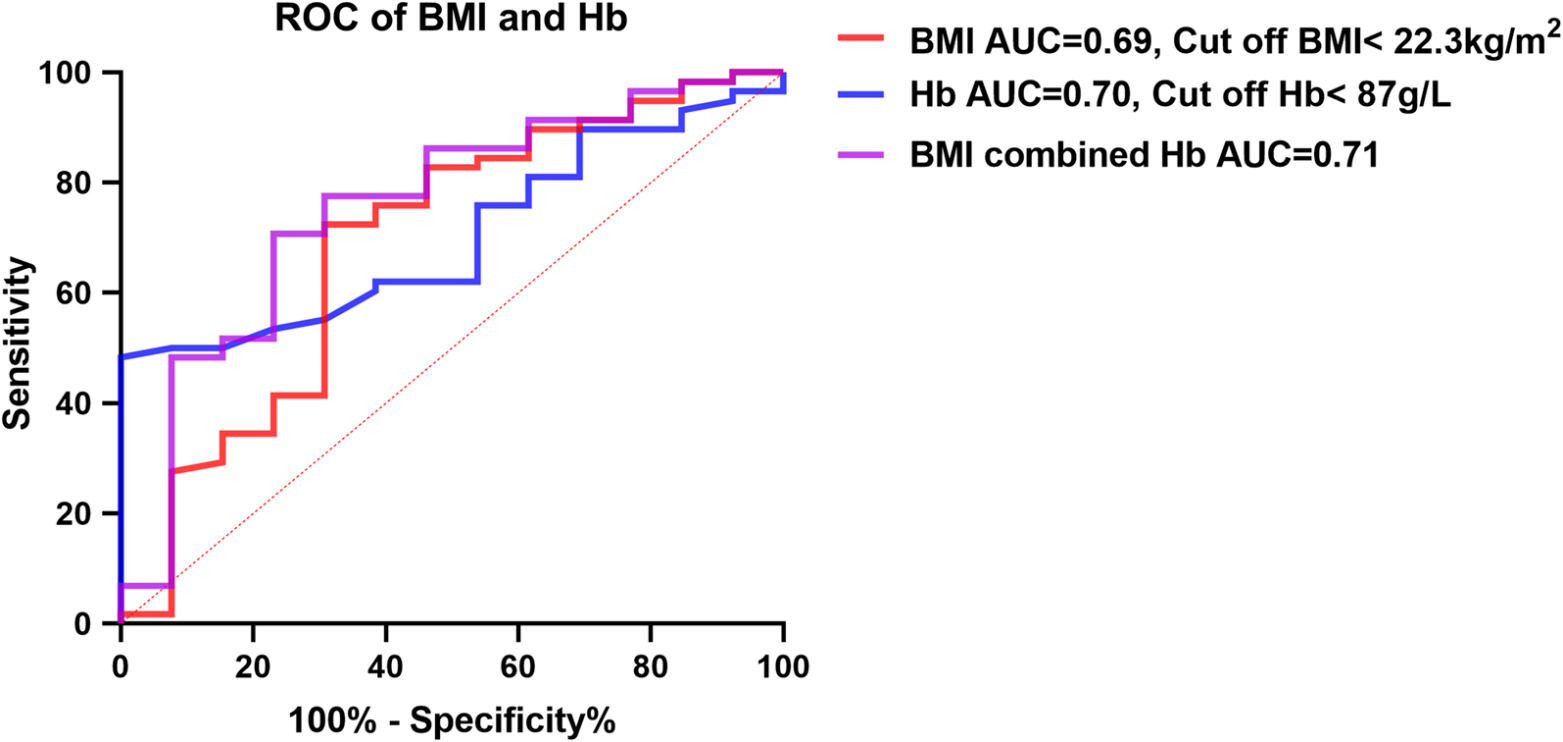
ROC Evaluation of the Predictive Value of BMI and Hb for CMV Reactivation

Furthermore, we plotted ROC curves for BMI and Hb levels to assess CMV reactivation’s predictive value. From Table 4 and Fig. 2, it could be seen that the AUC of BMI was 0.69 [Specificity (%): 72.4, Sensitivity (%): 69.2; 95% CI: 0.51–0.87; P = 0.03], Hb was 0.70 [Specificity (%): 48.3, Sensitivity (%): 100; 95% CI: 0.57–0.83; P = 0.02], and BMI combined with Hb was 0.76 [Specificity (%): 70.7, Sensitivity (%): 76.9; 95% CI: 0.60–0.91; P < 0.01]. We also used the cut-off method to obtain BMI < 22.3 kg/m^2^ as the threshold for predicting CMV reactivation, and Hb < 87 g/L as the threshold for predicting CMV reactivation.

**Table 4:** Predictive value of BMI and Hb on CMV reactivation

	AUC	Cut off	Specificity (%)	Sensitivity (%)	95% CI	*P*
BMI (kg/m^2^)^*a*^	0.69	22.3	72.4	69.2	0.51–0.87	0.03
Hb (g/L)^*a*^	0.70	87	48.3	100	0.57–0.83	0.02
BMI combined with Hb^*b*^	0.76	–	70.7	76.9	0.60–0.91	< 0.01

^a^P < 0.05; ^b^P < 0.01; *AUC* Area Under Curve, *95% CI* 95% Confidence Interval

### Clinical prognoses

**Fig. 3:**
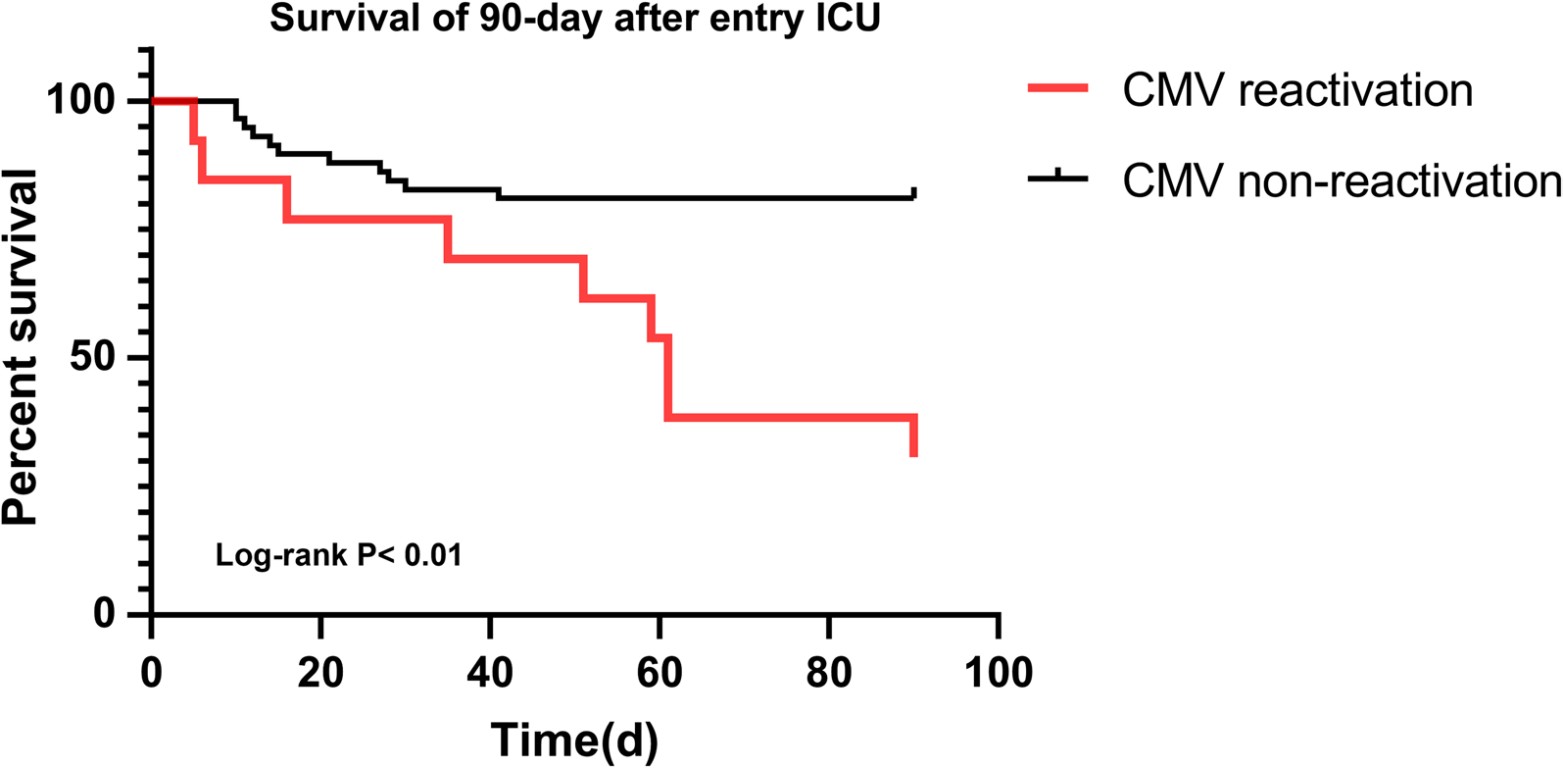
Kaplan–Meier Curves to Assess the Effect of CMV Reactivation on 90 day Survival after ICU Entry

It is clear from Table 5 that of 71 patients, 20 deceased within 90 days of ICU admission [90 day all-cause mortality: 28.2%], and non-survivors were more likely to be CMV reactivation cases compared with CMV non-reactivation cases [69.2% vs. 19.0% (n), P < 0.01]. As Fig. 3 shows, the Kaplan–Meier curve to evaluate patients’ 90 day survival was a significant difference [95% CI: 49.54–72.46, Log-rank P < 0.01] between the two groups. No evidence was found for 28 day mortality associations between CMV reactivation and non-reactivation groups.

**Table 5:** Clinical Outcomes of the Study Patients with and without CMV Reactivation

	Overall	CMV Reactivation		
	N = 71	Yes (n = 13, 18.3%)	No (n = 58, 81.7%)	*P*
Complications, n (%)^&a^	54 (76.1)	13 (100)	41 (70.7)	0.06
Blood Transfusion in ICU, n (%)	52 (73.2)	12 (92.3)	40 (69.0)	0.17
Length of IMV (d)^b^	**13 (8–20)**	**25 (20–45)**	**10 (8–18)**	** < 0.01**
Hospitalization Expenses (million/¥)^a^	**0.18 (0.11–0.26)**	**0.35 (0.35–0.23)**	**0.16 (0.11–0.23)**	**0.02**
Length of Hospital Stay (d)	29 (17–50)	50 (27–61)	28 (17–41)	0.10
ICU Length of Stay (d)^b^	**14 (9–20)**	**27 (20–45)**	**12 (8–18)**	** < 0.01**
28 day All-Cause Mortality, n (%)	17 (23.9)	5 (38.5)	12 (20.7)	0.32
90 day All-Cause Mortality, n (%)^b^	**20 (28.2)**	**9 (69.2)**	**11 (19.0)**	** < 0.01**

^a^P < 0.05; ^b^P < 0.01; Continuous variables were expressed as Mean ± SD or Median (IQRs); Bold font indicates the comparisons with statistical significance; ^&^At least one of the following complications—urinary tract infection, ventilator-associated pneumonia, gastric hemorrhage, disseminated intravascular coagulation, or acute heart failure; *IMV* Invasive Mechanical Ventilation

Significantly more CMV reactivation cases had length of IMV [median: 25 vs. 10 (days), P < 0.01], hospitalization expense [median: 0.35 vs. 0.16 (million/¥), P = 0.02] and length of ICU stay [median: 27 vs. 12 (days), P < 0.01]. The complication rate [100% vs. 70.7% (n), P = 0.06] was initiated more frequently in CMV reactivation patients than in non-reactivation patients. No significant differences were found between the rate of blood transfusion and length of hospital stay. Therefore, CMV reactivation is associated with several adverse clinical outcomes.

## Discussion

This study sought to investigate the incidence, risk factors, prognoses, and predictors of CMV reactivation in immunocompetent mechanical ventilation patients. We have found that, among immunocompetent patients requiring MV, the CMV seropositivity reached 100% and the incidence of reactivation was 18.3%. Clinical features, including BMI, sepsis, and biochemical indicators (NT-proBNP, BUN, Hb level) were related to CMV reactivation. Further statistical analysis showed that BMI, Hb, and sepsis had a moderate predictive value and were independent risk factors for CMV reactivation. In addition, CMV reactivation was associated with several adverse outcomes.

CMV reactivation has been a frequent phenomenon among patients admitted to the ICU. The positivity for CMV is 83% in the general population [1], although in our study, CMV seroprevalence was surprisingly found to be as high as 100%. This result may be related to the region (a developing country) and its poor health conditions. A 1990–2016 systematic review and meta-analysis review that included cohort studies estimated the incidence of CMV reactivation to be 31% in immunocompetent patients (95% CI, 24%–39%) in critical care settings [12]. In a prospectively observational study, which enrolled 120 patients with trauma, burns, and medical and cardiac issues, CMV DNAemia occurred at any level in 33% [13]. Another study, which enrolled 242 patients in a medical ICU and evaluated both blood and respiratory samples, indicated CMV reactivation in 16% of immunocompetent critically ill patients [14]. Furthermore, we summarized the relevant studies from 1990 to 2019 and found that the incidence of CMV reactivation was 0–71% based on CMV DNAemia in non-immunocompromised ICU patients [4, 8, 9]. Our finding of the lower incidence of CMV reactivation in patients with immunocompetent critically ill compared with most studies might have been attributable to ethnic and individual differences between study subjects. In particular, non-immunosuppressed mechanically ventilated critically ill patients were included in this study. Also, the detection methods (time points and monitoring periods) of CMV in different studies and the selection of different diseases as subjects may be related to the incidence of CMV reactivation. Moreover, it is important to note that this study used blood DNAemia, which might underestimate the CMV reactivation, combined with more comprehensive testing of airway specimens. Meanwhile, the finding that some study subjects had CMV reactivation within 24 h of ICU admission showed that this subgroup had CMV reactivation prior to ICU admission, so the clinical significance of CMV reactivation may not be limited to within the ICU stay.

Previous studies had demonstrated that sepsis was associated with CMV reactivation [4, 5, 15, 16], which was consistent with our results. Sepsis can induce CMV reactivation mainly through sepsis-related cytokine storm triggering transcriptional CMV replication, a mechanism that has been confirmed in animal models [4, 17, 18]. Several studies have shown that CMV infection can impair the cardiac and renal function, increasing cardiac and renal biochemical indicators [19, 20], which is consistent with this study’s results. The reason beyond this association is related to the direct pathological damage caused by CMV infection and indirect damage caused by inflammatory factors. Recent studies have shown that CMV infection can affect the body’s development and metabolic level and induce metabolic syndrome, which manifests as a chronic consumptive condition [21]. It can decrease lipids, BMI, and Hb in adults [22, 23]. Our study found that the BMI and Hb levels of the CMV reactivation group were lower, which may be related to CMV reactivation’s metabolic disorder. However, this speculation needs to be confirmed by more in vivo controlled trials.

Several studies revealed that CMV reactivation was strongly associated with sepsis, mechanical ventilation, and hypertension induced by glucocorticoids and catecholamines [24]. However, there was no correlation for disease scores, such as the APACHE and SOFA scores. Simultaneously, there was no evidence that CMV reactivation was age-related, but whether it was gender-related or not remained inconsistent. Besides, several clinical studies predicted CMV reactivation by cytokine levels (such as IL-6, IL-10, and TNF-α) [11, 14]. However, the current results were mostly negative, and no consistent results were found, which was similar to our study results. The reason may be related to the clinical condition’s complexity, and it is difficult to analyze the relationship between the immune system and CMV reactivation.

We found that BMI, Hb, and sepsis were independent risk factors for CMV reactivation through a multivariate logistic regression model and were independent of each other in the study population. It was further found that BMI and Hb levels had a moderate value for predicting CMV reactivation. There is no relevant study consistent with our results, and this association has never been studied before. BMI is a valid indicator that reflects the body nutrition level, a decline in which reflects body malnutrition and is associated with inflammation [25]. The inflammation storm induced by critical illness is a key trigger for CMV reactivation [4]; there is currently evidence of low BMI levels in patients infected with CMV [22, 24], which may help explain the relationship between BMI decline and CMV reactivation. However, more patients need to be included for further confirmation. Future studies are needed to further evaluate the relationship between different BMI levels (kg/m^2^: < 18.5, 18.5–25, 25–30, > 30) and the risk of CMV reactivation. In addition, the drop in hemoglobin may reflect the severity of the disease, due to the fact that critically ill patients often remain in a myelosuppressive state with subsequent loss of hematopoietic capacity and immune competence, while immunosuppression is associated with cytomegalovirus reactivation [4]. Moreover, CMV infection is one of the important myelosuppressive factors (mostly seen in bone marrow transplant patients) [26]. The above situations can lead to decreased hemoglobin levels and subsequently to increased transfusion risk, and it has been shown that transfusion is closely related to cytomegalovirus reactivation by several mechanisms, including direct factors (transmission of donor virus) and indirect factors (allogeneic stimulation) [27]. However, it needs to be emphasized that the sample size in this study was small, and there is a possibility of type II error.

Most of the findings suggested that CMV reactivation was related to the clinical prognoses of non-immunosuppressed patients, which is consistent with our findings, including prolonged duration of mechanical ventilation and ECMO, increased incidence of nosocomially acquired infections, and increased length of hospitalization and mortality [4–9, 11–15, 24, 28]. The causes of these adverse prognoses are various, including direct injury (such as CMV pneumonia) and indirect injury (such as immune disorder) [4]. Therefore, it is important for the treatment of CMV reactivation, but clinical trials about CMV reactivation prophylaxis did not evidently prove the clinical benefit [29, 30]. Recent preemptive therapeutic strategies for non-immunosuppressed mechanically ventilated patients with CMV reactivation to improve clinical outcomes remain controversial [31]. Preventive treatment strategies may be theoretically more meaningful because they prevent CMV reactivation and its associated direct or indirect damage. Antiviral therapy for specific disease species may be conducive to beneficial clinical outcomes.

Nonetheless, this study has several limitations. First, this study was a single-center observational study. The number of patients included was relatively insufficient to comprehensively evaluate CMV’s epidemiological characteristics with immunocompetent mechanical ventilation patients. Second, the number of patients with sepsis was insufficient for subgroup analysis, though sepsis patients may present a higher incidence of CMV reactivation. Third, some patients had CMV reactivation on the day of ICU admission, presumably before ICU admission, which might impact outcomes. Therefore, prospective, multicenter studies are needed in the future, and more subjects with sepsis should be included. Meanwhile, the observation of CMV reactivation needs to be extended to the entire hospitalization period and not limited to the ICU stay period.

## Conclusions

The incidence of CMV reactivation was 18.3% on the immunocompetent mechanical ventilation patients. Furthermore, CMV reactivation was associated with prolonged IMV, increased hospitalization expenses, prolonged ICU hospitalization, and increased 90 day mortality. CMV reactivation was also related to higher rates of transfusion and complications. BMI, Hb, and sepsis were independent risk factors for CMV reactivation. BMI and Hb may predict CMV reactivation.

## Supplementary Information

**Additional file 1. Figure S1. **Incidence of CMV Reactivation within 28 day Hospitalization in ICU. **Figure S2. **Time of CMV Reactivation within 28 day Hospitalization in ICU. **Figure S3.** DNAemia of CMV Reactivation within 28 day Hospitalization in ICU. **Table S1.** Immune Indicators of the Study Patients at the Time of ICU Admission.

## Data Availability

Data sharing will be considered only on a collaborative basis with the principal investigators, after evaluation of the proposed study protocol and statistical analysis plan.

## Abbreviations

CMV: Cytomegalovirus
ICU: Intensive Care Unit
ARDS: Acute Respiratory Distress Syndrome
AKI: Acute Kidney Injury
MV: Mechanical Ventilation
IMV: Invasive Mechanical Ventilation
BMI: Body Mass Index
ECMO: Extracorporeal Membrane Oxygenation
APACHE II: Acute Physiology and Chronic Health Evaluation
SOFA: Sequential Organ Failure Assessment
P/F: PaO_2_/FiO_2_
QRT-PCR: Quantitative Real-time Polymerase Chain Reaction
COPD: Chronic Obstructive Pulmonary Disease
AECOPD: Acute Exacerbation of Chronic Obstructive Pulmonary Disease
SPO_2_: Arterial Oxygen Saturation
Th1/Th2: Helper T Lymphocyte 1&2
PT: Prothrombin Time
APTT: Activated Partial Thromboplastin Time
NT-proBNP: N-terminal Pro-B-type Natriuretic Peptide
AST: Aspartate Aminotransferase
ALT: Alanine Transaminase
T-BIL: Total Bilirubin
Scr: Serum Creatinine
BUN: Blood Urea Nitrogen
Hb: Hemoglobin
PCT: Procalcitonin
CRP: C-reactive Protein
ESR: Erythrocyte Sedimentation Rate
NK: Natural Killer
Ig: Immunoglobulin
C: Complement
IL: Interleukin
INF: Interferon
TNF: Tumor Necrosis Factor
GM-CSF: Granulocyte–macrophage Colony Stimulating Factor
IQRs: Interquartile Ranges
β: Regression Coefficient
OR: Odds Ratio
ROC: Receiver Operating Curve
AUC: Area Under Curve
CI: 95% Confidence Interval
KM: Kaplan–Meier
HR: Hazard Ratio

## Declarations

### Statement

All methods were carried out in accordance with relevant guidelines and regulations in the manuscript.
