# Supplementary material for: Cytomegalovirus reactivation in immunocompetent mechanical ventilation patients: a prospective observational study
Source: BMC Infect Dis. 2021 Sep 30;21:1026. doi: 10.1186/s12879-021-06698-0 (PMC8482357; doi:10.1186/s12879-021-06698-0)
Supplement: Supplementary file 1 — Additional file 1. Figure S1. Incidence of CMV Reactivation within 28 day Hospitalization in ICU. Figure S2. Time of CMV Reactivation within 28 day Hospitalization in ICU. Figure S3. DNAemia of CMV Reactivation within 28 day Hospitalization in ICU. Table S1. Immune Indicators of the Study Patients at the Time of ICU Admission. [file 12879_2021_6698_MOESM1_ESM.doc]

**Additional file 1 (Results)**

**Cytomegalovirus Reactivation in Immunocompetent Mechanical Ventilation Patients: A Prospective Observational Study**

**Results**

**Viral load of CMV reactivation**

CMV DNAemia showed CMV had different viral loads in different patients. Among the 13 patients who showed CMV reactivation within 28 days in the ICU, 8 (61.5%) had 2001–3000 copies/mL, 3 (23.1%) had 3001–4000 copies/mL, and 2 (15.4%) had > 5000 copies/mL, shown in **Figure S3**.


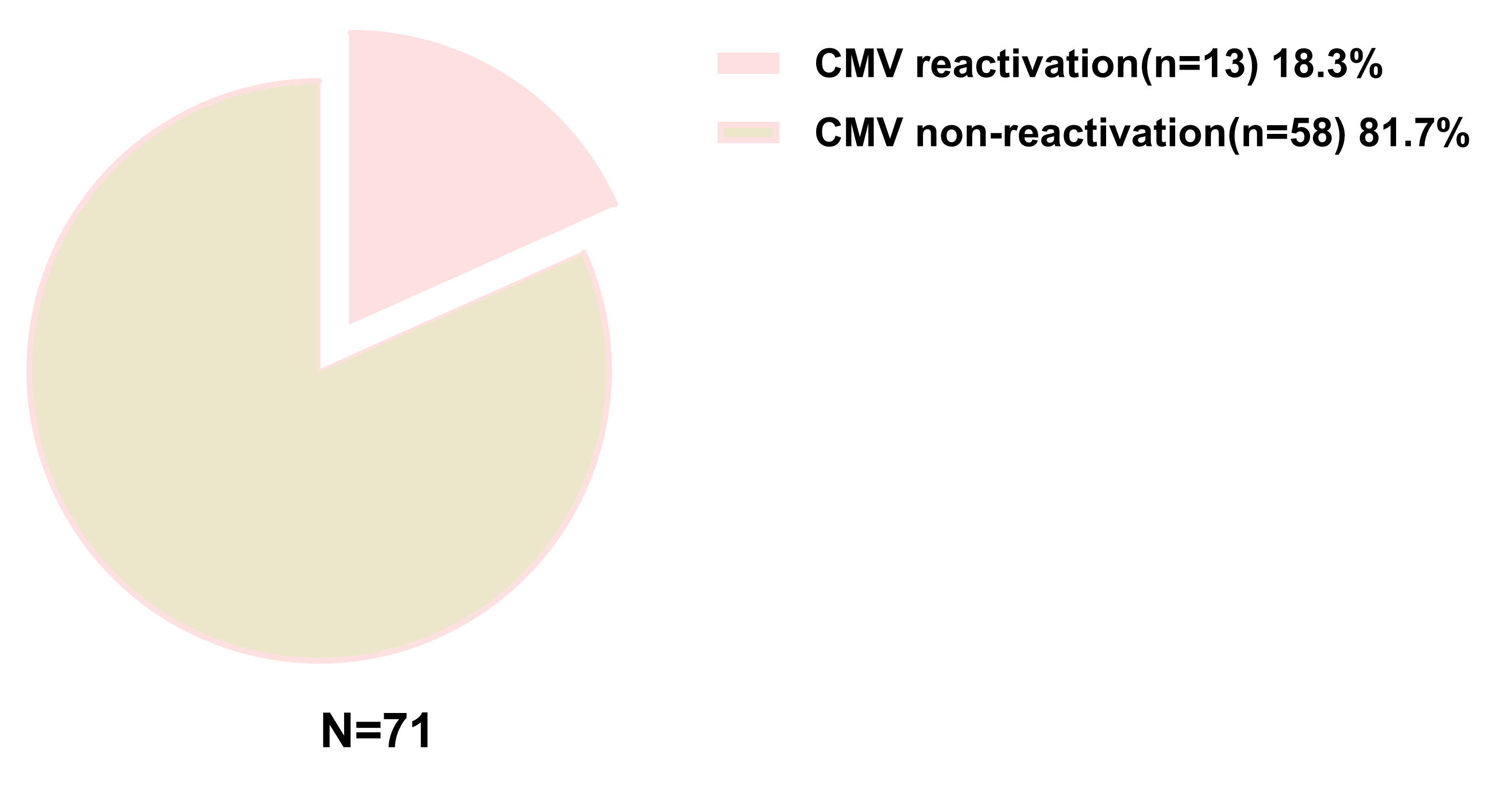


**Figure S1. Incidence of CMV Reactivation within 28-day Hospitalization in ICU.**


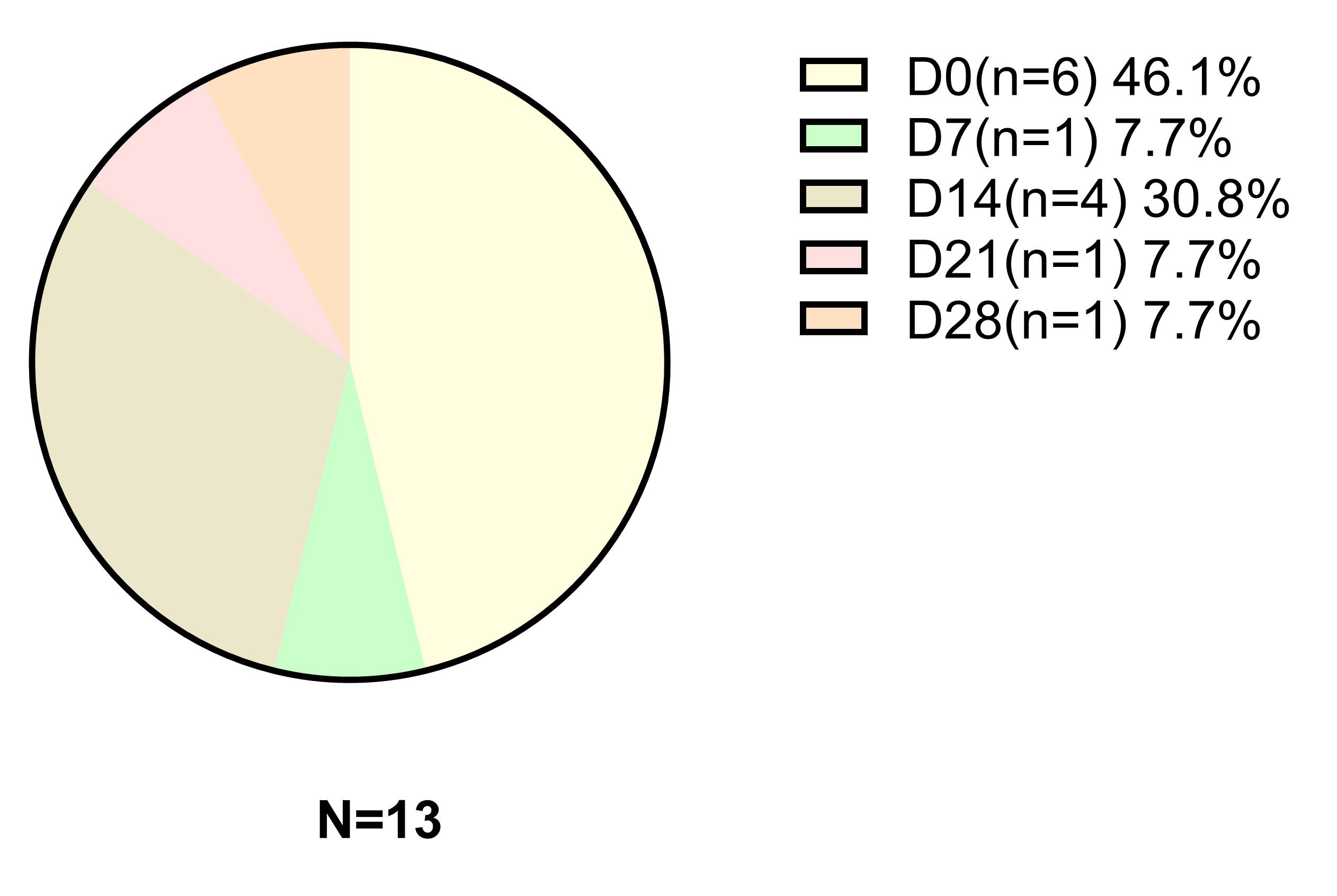


**Figure S2. Time of CMV Reactivation within 28-day Hospitalization in ICU.**


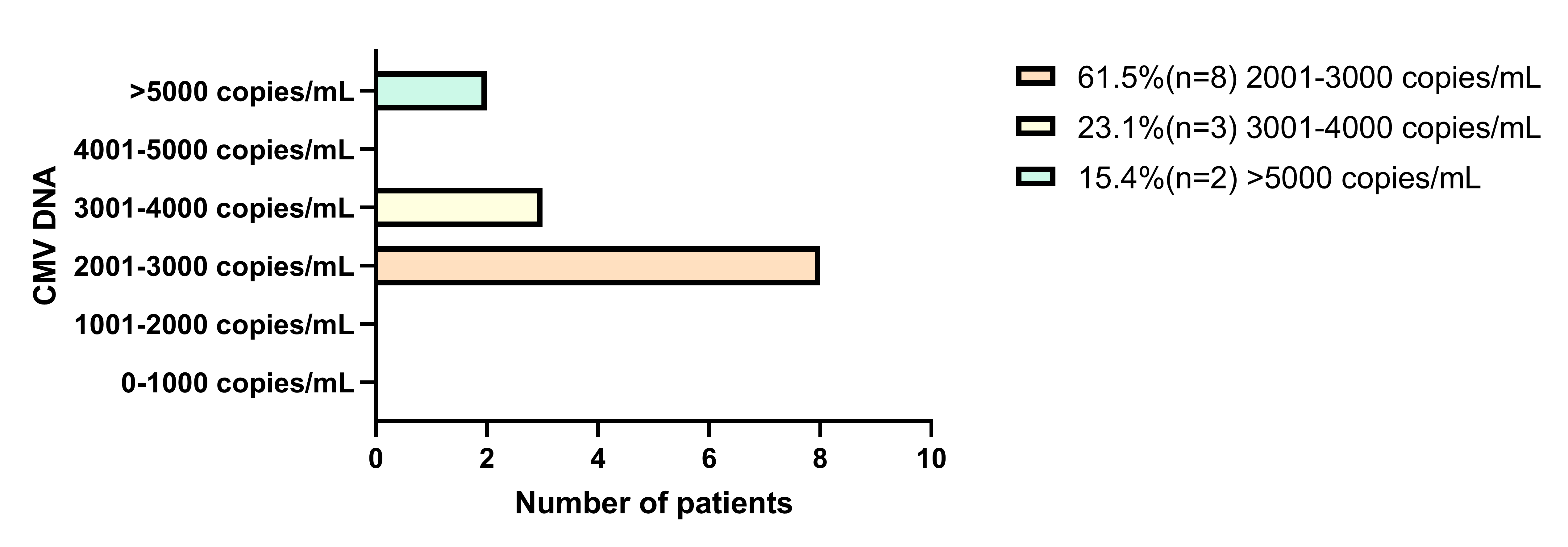


**Figure S3. DNAemia of CMV Reactivation within 28-day Hospitalization in ICU.**

**Table S1. Immune Indicators of the Study Patients at the Time of ICU Admission.**

|  | **Overall** | **CMV Reactivation** | | |  |
| --- | --- | --- | --- | --- | --- |
|  | **N=71** | **Yes**  **(n=13, 18.3%)** | **No**  **(n=58, 81.7%)** | | ***P*** |
| **Lymphocyte Subpopulations (%)** |  |  | |  |  |
| T Lymphocytes | 64.4 ± 12.8 | 63.1 ± 17.1 | | 64.7 ± 11.7 | 0.74 |
| Th | 39.2 ± 11.4 | 39.7 ± 13.8 | | 39.1 ± 11.0 | 0.88 |
| Ts | 19.5 (16.8-25.7) | 19.9 (16.8-24.9) | | 19.5 (16.8-25.7) | 0.90 |
| Th/Ts | 1.9 (1.4-2.6) | 1.9 (1.4-2.7) | | 1.9 (1.5-2.6) | 0.99 |
| B Lymphocytes | 19.2 ± 11.8 | 24.5 ± 16.8 | | 18.0 ± 10.2 | 0.21 |
| NK | 13.9 ± 10.2 | 10.3 ± 6.7 | | 14.7 ± 10.7 | 0.07 |
| **Immune Molecules (g/L)** |  |  | |  |  |
| IgG | 9.7 (8.0-12.6) | 8.5 (7.4-11.7) | | 9.9 (8.6-13.0) | 0.18 |
| IgA | 1.8 (1.2-3.1) | 1.4 (1.1-2.3) | | 1.9 (1.4-3.1) | 0.15 |
| IgM | 0.7 (0.5-0.9) | 0.6 (0.4-0.7) | | 0.7 (0.5-0.9) | 0.20 |
| C3 | 0.7 (0.5-0.8) | 0.7 (0.5-0.8) | | 0.7 (0.5-0.8) | 0.99 |
| C4 | 0.2 ± 0.2 | 0.2 ± 0.1 | | 0.2 ± 0.1 | 0.58 |
| **Th1/Th2 Cytokines (****mmol/L)** |  |  | |  |  |
| IL-2 | 10.3 (6.5-12.8) | 10.5 (7.4-13.4) | | 10.3 (6.5-12.8) | 0.77 |
| IL-4 | 2.2 (1.7-2.7) | 2.2 (1.5-2.7) | | 2.3 (1.7-2.7) | 0.60 |
| IL-6 | 5.4 (2.9-7.3) | 5.4 (3.7-6.7) | | 5.2 (2.9-7.3) | 0.75 |
| IL-8 | 26.1 (17.6-46.7) | 42.2 (22.6-60.4) | | 26.1 (17.6-46.0) | 0.30 |
| IFN-γ | 2.9 (2.3-4.1) | 2.9 (2.3-3.2) | | 2.7 (2.3-4.1) | 0.98 |
| TNF-α | 40.9 (31.9-53.5) | 47.8 (31.9-62.8) | | 40.1 (31.9-51.9) | 0.30 |
| GM-CSF | 2.6 (2.4-2.9) | 2.6 (2.6-2.6) | | 2.6 (2.4-3.1) | 0.56 |

***Continuous variables were expressed as Mean ± SD or Median (IQRs). Th: T-helper Lymphocytes; Ts: T-suppressor Lymphocytes; NK: Natural Killer; Ig: Immunoglobulin; C: Complement; IL: Interleukin; INF: Interferon; TNF: Tumor Necrosis Factor; GM-CSF: Granulocyte-macrophage Colony Stimulating Factor.***
